# Retroperitoneal schwannoma mimicking a metastatic lymph node of renal clear cell carcinoma: a case report

**DOI:** 10.3389/fneur.2024.1450217

**Published:** 2024-08-02

**Authors:** Shaorong Pan, Pengyuan Wang, Zeyang Chen, Yucun Liu, Zhengfei Zhou

**Affiliations:** ^1^Department of Gastrointestinal Surgery, Peking University First Hospital, Peking University, Beijing, China; ^2^Department of Hepatobiliary and Pancreatic Surgery, Peking University First Hospital, Peking University, Beijing, China

**Keywords:** schwannoma, renal clear cell carcinoma, metastatic lymph node, radiological examination, case report

## Abstract

Schwannomas are usually benign tumors typically found in the head, neck, and extremities, with approximately 3% originating in the retroperitoneum. In this case, a young male presented with incidental masses in the left kidney and retroperitoneum. Abdominal pelvic enhanced computerized tomography (CT) revealed a tumor apparently originating from the left kidney, along with a retroperitoneal mass suspected to be a metastatic lymph node. Subsequently, a radical nephrectomy of the left kidney and retroperitoneal mass resection was performed. Pathological examination confirmed the left kidney mass as renal clear cell carcinoma and the retroperitoneal mass as schwannoma. The patient recovered uneventfully and was discharged from the hospital. A 6-month postoperative follow-up showed no evidence of recurrence. Preoperative diagnosis of schwannomas concurrent with other concurrent malignancies in rare sites, such as the retroperitoneum, is challenging due to their rare and non-specific radiological features. Although retroperitoneal schwannomas are rare, they should be considered in the differential diagnosis during CT examinations for renal cancer. Additionally, the advantages of a multidisciplinary team approach should be utilized in tumor management.

## 1 Introduction

Schwannomas are typically benign tumors that develop from Schwann cells in the peripheral nerve sheath, most commonly found in the head, neck, and extremities, with approximately 3% originating in the retroperitoneum (1). Most schwannomas present as asymptomatic or painless masses and are often discovered incidentally during radiological examinations. Due to their rarity and non-specific radiological features, preoperative diagnosis is challenging, especially when other malignancies are present at the same time. Here we present a case of retroperitoneal schwannoma complicated by renal clear cell carcinoma. The radiological characteristics of the retroperitoneal schwannoma in our case were similar to those of metastatic lymph nodes from renal cancer, complicating the preoperative staging and surgical treatment plan. We hope this case provides valuable insights and reference for clinicians encountering similar scenarios.

## 2 Case presentation

**Figure 1:**
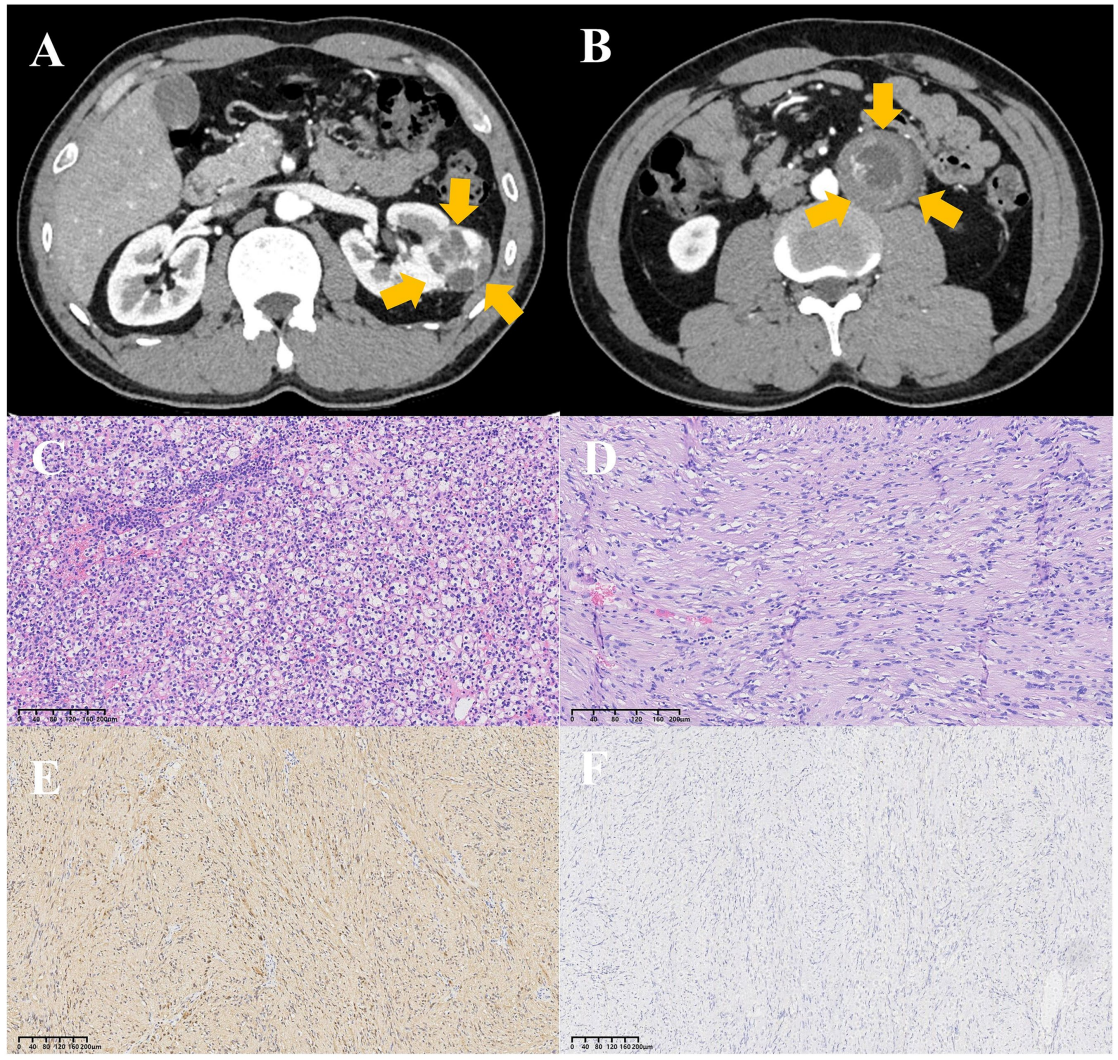
Abdominal pelvic enhanced computerized tomography (CT) and pathology of the left kidney and retroperitoneal masses. **(A)** Heterogeneously enhancing mass in the middle of the left kidney with necrotic cysts and bleeding (yellow arrows). **(B)** Non-uniform enhanced mass with central necrosis in the retroperitoneum (yellow arrows). **(C)** Renal clear cell carcinoma of the left renal mass. **(D)** Schwannoma of the retroperitoneal mass. **(E)** Retroperitoneal mass staining strongly positive for S-100 on immunohistochemistry (IHC). **(F)** Retroperitoneal mass staining strongly negative for CD117 on IHC.

A 39-year-old man without significant clinical signs, symptoms, past medical history and familial-hereditary disease was found to have masses in the middle of the left kidney and retroperitoneum during a routine physical examination. Except for a slight increase in neuron-specific enolase (17.15 ng/mL, normal range <16.3 ng/mL), his laboratory tests showed no significant abnormalities. Abdominal pelvic enhanced computerized tomography (CT) revealed a 3.9 cm*3.7 cm*3.3 cm protuberant mass with a rich blood supply in the middle of the left kidney, containing necrotic cysts and hemorrhage (Figure 1A). The findings were highly suggestive of renal cancer, with perirenal fat invasion, although it did not exceed the perirenal fascia. Additionally, a 4.7 cm*4.5 cm*3.8 cm heterogeneous enhanced retroperitoneal mass with central necrosis was observed, considered to be metastatic lymph nodes (Figure 1B). Preoperative imaging of this retroperitoneal mass suggested lymph node metastasis of renal cancer which is not routinely diagnosed by puncture biopsy. Moreover, since the mass appeared to be an isolated “metastasis” with the potential for surgical resection, a preoperative biopsy was deemed unnecessary. Additionally, certain benign renal tumors, such as eosinocytomas, can closely mimic renal cancer under microscopic examination. A small tissue sample from the puncture biopsy might not provide sufficient information for pathologists to accurately determine the biological characteristics of tumor. Therefore, it is crucial to send the entire tumor for pathological examination to ensure an accurate diagnosis. Besides, the retroperitoneal location of the tumor increases the risk of complications from a puncture biopsy, such as damage to surrounding structures like blood vessels and intestines, which can lead to bleeding, infection and needle tract metastasis. Given these considerations, our multidisciplinary team (MDT) comprising urologists, general surgeons and radiologists decided to perform a radical nephrectomy of the left kidney and resection of the retroperitoneal mass without a preoperative puncture biopsy. The operation was completed successfully.

Postoperative pathology revealed that the left renal mass was renal clear cell carcinoma (Figure 1C), graded as International Society of Urological Pathology (ISUP) grade 2. The renal pelvis, renal sinus fat and renal capsule were not involved, and there was no evidence of vascular tumor embolus or nerve invasion. The surgical margins of the vascular and ureteral stumps were negative. Pathological examination of the retroperitoneal mass showed spindle cell tumors with an uneven distribution of cells under microscope (Figure 1D). In densely cellular areas, the tumor cells were arranged in bundles or mat patterns, while in some area, the tumor cells were sparse with visible mucoid stroma. The cell morphology was mild, and no mitotic figures was observed. Immunohistochemistry (IHC) demonstrated positive staining for S-100 (Figure 1E) while CD117 (Figure 1F), CD34 and DOG-1 were negative. Based on these findings, the final pathological diagnosis of the retroperitoneal mass was schwannoma.

The patient recovered well, achieving normal eating and mobility within 2 weeks post-operation, and was subsequently discharged successfully. Both the patient and his family expressed satisfaction with the treatment and have been undergoing regular follow-up evaluations. At the 6-month follow-up, the patient remained alive and free from disease recurrence.

## 3 Discussion

Schwannoma is the most common peripheral nerve tumor, consisting entirely of neoplastic Schwann cells (2, 3). Most schwannomas are benign, with malignant transformation being rare (4, 5). These tumors originate from peripheral nerves or nerve roots and typically grow eccentrically, with the nerves usually encased within a capsule. The vast majority of schwannomas are solitary and sporadic, but they can also be associated with certain conditions, presenting as multiple schwannomas in diseases such as neurofibromatosis type 2 (NF2), schwannomatosis, Carney syndrome, and syndrome with nevi and vaginal leiomyomas (6). Schwannomas are not associated with neurofibromatosis type 1 (NF1). Neurofibromatosis (NF) is an autosomal dominant genetic disorder characterized by multisystem damage due to abnormal neural crest cell development resulting from genetic defects. It primarily manifests as benign tumor growth within the nervous system. Based on their molecular characteristics, NF is classified into NF1, NF2, and schwannomatosis. NF2 and schwannomatosis are often associated with multiple schwannomas. In contrast, the hallmark clinical features of NF1 include Café-au-lait macules on the skin and the presence of multiple neurofibromas or plexiform neurofibromas, without the presence of schwannomas.

Solitary schwannomas can affect individuals of all ages, with the peak incidence occurring between the ages of 20 and 50, regardless of gender or ethnicity (3). These tumors commonly arise from the peripheral nerves of the head, neck, and flexor surfaces of the extremities and although they can also occur in the mediastinum, retroperitoneal schwannomas are rare. Many schwannomas are discovered incidentally as solitary tumors on the flexor surfaces of middle-aged individuals (5). Our literature search identified approximately 30 reported cases of renal schwannomas (1), with only a few cases of retroperitoneal schwannomas complicated by renal oncocytoma or hybrid oncocytic/chromophobe tumors (7, 8). Additionally, schwannomas in the porta hepatis complicated by renal clear cell carcinoma have also been reported (9). To our knowledge, our case represents the first report of retroperitoneal schwannomas accompanied by renal clear cell carcinoma. Although the coexistence of renal cancer and schwannoma is rare, some studies have suggested a potential genetic relationship between the two conditions. The loss of function of merlin (also known as schwannomin) has been proposed as a possible explanation for the occurrence of both sporadic and genetically acquired schwannomas (10). Furthermore, genetic alterations in *NF2* (the gene encoding merlin) have been implicated in a subtype of renal cell carcinoma known as collecting duct carcinoma (11). However, more cases are needed to provide molecular evidence for a definitive link between these conditions.

Most individuals with schwannomas are asymptomatic, but they may experience paresthesia (often induced by palpation), radicular pain, anesthesia, and muscle weakness, typically resulting from the compression of adjacent nerve structures such as the brachial plexus (12). Schwannomas can also be incidentally detected through radiological examination as in our case.

Schwannomas exhibit a biphasic structure characterized by fasciculate (Antoni A, dense) and reticular (Antoni B, loose) patterns, with palisading nuclei forming Verocay bodies and fibrous sacs containing the originating nerves (2). Neoplastic Schwann cells typically possess fusiform nuclei. Pathological variations of schwannomas include cellular, melanotic and plexiform types (2). Cellular schwannomas primarily consist of fascicular tissues without Verocay bodies. Melanotic schwannomas exhibit dense melanosis but otherwise conform to typical schwannoma morphology. Psammomatous melanotic schwannoma, a subtype of melanotic schwannomas, is distinguished by the presence of psammoma bodies (rounded, layered calcium deposits). Notably, melanotic schwannomas carry a potential for malignant transformation unlike other schwannomas. Plexiform schwannomas, although rare, usually manifest along the nerve plexus as multiple schwannomas clusters and may be sporadic or associated with NF2-related schwannomatosis and other forms of schwannomatosis. Unlike plexiform neurofibroma, plexiform schwannomas do not undergo malignant transformation. In addition, persistent schwannomas may undergo degeneration, exhibiting significant nuclear pleomorphism, extensive vascular hyalinoid degeneration, evidence of remote hemorrhage, focal necrosis, and calcification. Historically referred to as “ancient schwannomas” (3), these tumors are not considered clinically unique by many pathologists and are not typically recognized as a distinct schwannoma variant. The schwannoma in our case, featuring central necrosis, falls into this category. Key distinguishing features of schwannomas include the presence of a capsule, Antoni A and B regions, and uniform, strong S-100 protein immunostaining, differentiating them from neurofibromas. Schwannoma also expresses glial fibrillary acidic protein (GFAP) and CD57 (Leu 7), but lack expression of CD117, CD34, and DOG-1, markers which can help differentiate them from certain gastrointestinal tumors that may also stain positive for S-100 (13, 14).

Schwannomas typically exhibit lower density than muscles on CT scans, with notable enhancement after contrast-enhanced scanning (3). On T1-weighted magnetic resonance imaging (MRI), schwannomas show moderate signal intensity akin to muscle, whereas on T2-weighted images they present with high signal intensity (3, 15). These tumors generally show significant enhancement with approximately 70% displaying a low-signal edge indicative of a capsule. T2-weighted and contrast-enhanced images often reveal a fusiform structure and a target sign. Despite these features, schwannomas lack specific CT or MRI characteristics (16). Positron emission tomography (PET) scans of schwannomas reveal high metabolic uptake, potentially leading to misdiagnosis as primary malignant or metastatic lesions (17). In our case, the schwannoma was retroperitoneal and coexisted with renal carcinoma. Its CT findings showed heterogeneous enhancement with central necrosis, closely resembling the radiological features of metastatic lymph nodes of renal cancer. This similarity can lead to preoperative misdiagnosis as metastatic lymph nodes, thereby affecting the staging of renal cancer and influencing treatment plans.

Although schwannomas are the most common peripheral nerve tumors, the surgical resection rate due to symptoms is only about half that of neurofibromas. However, preoperative diagnosis of schwannomas concurrent with other malignancies, especially in rare locations like the retroperitoneum, is challenging owning to their rarity, nonspecific radiological features, and fluorodeoxyglucose (FDG) uptake on PET scans. Surgical resection remains the primary treatment method, and recurrence is rare even after partial resection.

Renal clear cell carcinoma complicated by retroperitoneal schwannoma is a rare clinical occurrence. In this case report, we detailed the differential diagnosis of these conditions from both imaging and pathological perspectives. However, certain limitations exist in this case report. Specifically, the lack of genetic testing on the surgically resected specimens prevents us from providing clinical evidence of a genetic relationship between the two tumors. However, this limitation highlights an important area for future research.

## 4 Conclusion

With the continuous development of radiological techniques, clinicians increasingly rely on radiological examinations for diagnosis, staging, treatment planning, and prognostic evaluation. However, one of the limitations of radiology is the potential for benign tumors to be misidentified as malignant lesions, which can significantly impact treatment strategies and patient psychology. Our case underscores these complexities, demonstrating that benign tumors such as schwannomas can exhibit radiological features on CT scans similar to those of malignant lesions. Despite their rarity, retroperitoneal schwannomas should be included in the differential diagnosis during CT examination for renal cancer. This case also highlights the importance and benefits of a multidisciplinary team approach in cancer management.

## Data availability statement

The original contributions presented in the study are included in the article/supplementary material. Further inquiries can be directed to the corresponding authors.

## Ethics statement

Written informed consent was obtained from the individual(s) for the publication of any potentially identifiable images or data included in this article.

## Author contributions

SP: Conceptualization, Data curation, Formal analysis, Investigation, Methodology, Software, Visualization, Writing – original draft, Writing – review & editing. PW: Investigation, Validation, Writing – original draft. ZC: Investigation, Validation, Writing – original draft. YL: Funding acquisition, Project administration, Resources, Supervision, Validation, Writing – review & editing. ZZ: Funding acquisition, Project administration, Resources, Supervision, Validation, Writing – review & editing.
